# Cluster-assembled metallic glasses

**DOI:** 10.1186/1556-276X-8-339

**Published:** 2013-07-30

**Authors:** Aras Kartouzian

**Affiliations:** 1 Chemistry Department, Chair of Physical Chemistry, Technical University of Munich, Lichtenbergstraβe 4, Garching 85748, Germany

**Keywords:** Bottom-up approach, Metallic glasses, Metal clusters

## Abstract

A bottom-up approach to nanofabricate metallic glasses from metal clusters as building blocks is presented. Considering metallic glasses as a subclass of cluster-assembled materials, the relation between the two lively fields of metal clusters and metallic glasses is pointed out. Deposition of selected clusters or collections of them, generated by state-of-the-art cluster beam sources, could lead to the production of a well-defined amorphous material. In contrast to rapidly quenched glasses where only the composition of the glass can be controlled, in cluster-assembled glasses, one can precisely control the structural building blocks. Comparing properties of glasses with similar compositions but differing in building blocks and therefore different in structure will facilitate the study of structure–property correlation in metallic glasses. This bottom-up method provides a novel alternative path to the synthesis of glassy alloys and will contribute to improving fundamental understanding in the field of metallic glasses. It may even permit the production of glassy materials for alloys that cannot be quenched rapidly enough to circumvent crystallization. Additionally, gaining deeper insight into the parameters governing the structure–property relation in metallic glasses can have a great impact on understanding and design of other cluster-assembled materials.

## Background

Metal clusters have been the subject of intensive investigations in the last three decades not only because they exhibit fascinating properties that largely differ from their atomic and bulk counterparts but also their size dependence and structure dependence provide unthinkable possibilities. Addition of a single atom may cause property alteration of appreciable magnitude [1-5]. Although metal clusters possess unique properties, the majority of their properties are not harvested mainly due to their high sensitivity to the surrounding environment. Metal clusters are usually produced and investigated under ultra-high vacuum conditions, which are hardly applicable outside modern research laboratories. Many innovative scientists have spelled out the desire to fabricate a new class of materials that are built from atomic clusters instead of individual atoms, in order to benefit from the unique properties of such clusters. In this respect, some examples are already realized [6-8] as so-called cluster-assembled materials (CAM).

Metallic glasses (MG) have also been studied extensively since the first amorphous metallic alloy was introduced more than half a century ago. By cooling with a high rate, Klement et al. observed the formation of glassy structure in a binary alloy Au_75_Si_25_[9]. They also reported the instability of this material at room temperature. After discovery of bulk metallic glasses and hence the possibility to create amorphous structures with moderate cooling rates, various multicomponent alloys were found with high glass-forming ability. Many of these alloys are usable under normal conditions, and several industrial applications are currently realized [10-14]. Despite the intensive research in the field of MGs, the fundamental question about the correlation between their structure and their unique properties is yet to be answered. The major challenge to this end is rooted in the lack of a descriptive model for the structure of MGs. So far, their structures are merely considered as a collection of atoms without long-range order in contrast to crystalline materials. This definition fails to distinguish among various amorphous materials and leaves the separation to the composition of alloys.

Cluster-based models such as efficient cluster packing, cluster-plus-glue atom, and cluster resonance have already been suggested to describe the arrangement of atoms in metallic glasses. Many research groups have demonstrated the appositeness of these models through theoretical simulations in combination with experimental structure analysis [15-39]. In this context, metallic glasses are considered as a subcategory of CAMs.

Here, nanofabrication of metallic glasses through the bottom-up approach incorporating size-controlled metallic clusters is proposed.

## Presentation of the hypothesis

**Figure 1:**
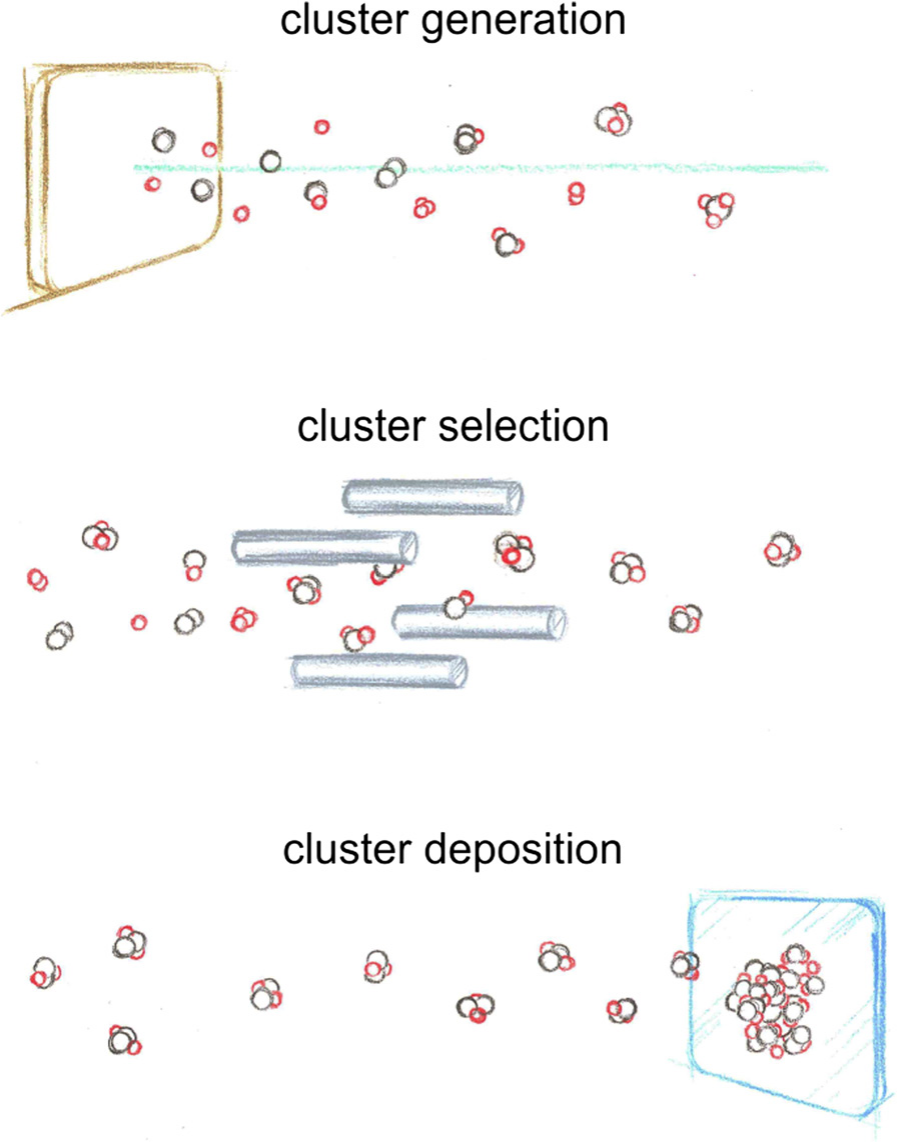
**Bottom-up approach to nanofabrication of metallic glasses.** (Top) Mixed metal clusters are generated by laser vaporization of a metal alloy target. (Middle) Using mass selection, a specific cluster is picked out of the cluster beam. (Bottom) Mass-selected clusters are deposited on a support material to form a metallic film.

Metal clusters of various compositions and sizes can be produced by a state-of-the-art cluster beam source. Recent advances in the field of cluster science enable us to overcome the quantity gap and create a well-defined cluster films of several monolayer thickness with atomic precision within few hours. Interestingly, altering the set of the mass-selected clusters while keeping the overall composition the same would lead to the formation of a potentially different material. For example, a Cu_0.5_Zr_0.5_ film can be fabricated by deposition of CuZr dimers, Cu_2_Zr_2_ tetramers, or equal numbers of Cu_6_Zr_7_ and Cu_7_Zr_6_ clusters just to name some of the numerous possibilities. All these films have the same composition and, however, different structures. A schematic view of the sample preparation approach is depicted in Figure 1. The structure and local atomic structure of the film can be explored by surface X-ray diffraction and extended X-ray absorption fine structure experiments, respectively. Electron microscopy may also be employed for similar studies. Valuable insight could be gained by comparing the properties of the cluster films with known building blocks to metallic glasses with similar composition, which are created via conventional methods such as rapid quenching, melt spinning, and ball milling. The first aim at this stage would be to explore the experimental conditions under which the structural properties of the cluster film are closest to the corresponding metallic glass. This would allow correlating the properties of the MG to its structure due to the available knowledge of its building blocks.

## Testing of the hypothesis

The first experiment of the kind should be performed on CuZr system based on the following reasoning. This system has been the subject of many experimental and theoretical studies in the past. Consequently, much is known about this binary system. Since only two metals are involved, generation of suitable binary clusters and their mass selection is easier compared to other multicomponent systems. In addition, CuZr alloys are known to be good glass formers over a range of compositions with glass transition temperature well above the room temperature [40-42]. The fact that both elements appear in more than one stable isotope, however, counts as a drawback. This makes the mass selection and cluster isolation more challenging.

Binary metal clusters can be generated using alloy targets. Ion beam techniques employed in the production of the metal clusters facilitate the use of high-resolution size selection filters. On the basis of the recorded mass spectra, the most intense mixed cluster should be isolated and deposited on a support material, which is kept at a temperature low enough to avoid crystallization of the film during deposition. It is expected that clusters with 13 atoms (Cu_m_Zr_n_, *n* + *m* = 13) form icosahedra and thus benefit from enhanced structural stability. The composition of the most abundant mixed cluster may vary for different cluster sources and with source conditions. Particular care should be taken to avoid oxidation of metal clusters prior and during deposition. To assure the latter, cluster deposition should be performed under ultra-high vacuum conditions. Finally, the sample should be handled under controlled environment (e.g., inert gas) and below room temperature (to avoid postdeposition oxidation and crystallization) throughout the analysis process. The properties of the specific metal cluster or clusters (if a combination of them is used to produce the cluster film) can be investigated to gain knowledge on the structural building blocks. The optical, electronic, geometric, magnetic, and binding energies of metal clusters can be determined both theoretically and experimentally by state-of-the-art scientific instruments. In parallel experiments, a film of conventional metallic glass prepared through rapid quenching processes but with an identical composition as cluster film should be analyzed for comparison purposes. A constructive feedback loop between these two types of metallic glasses synthesized through bottom-up approach and conventional methods is of great importance to unravel fundamental uncertainties associated with structure-dependent properties of metallic glasses.

## Implication of the hypothesis

**Figure 2:**
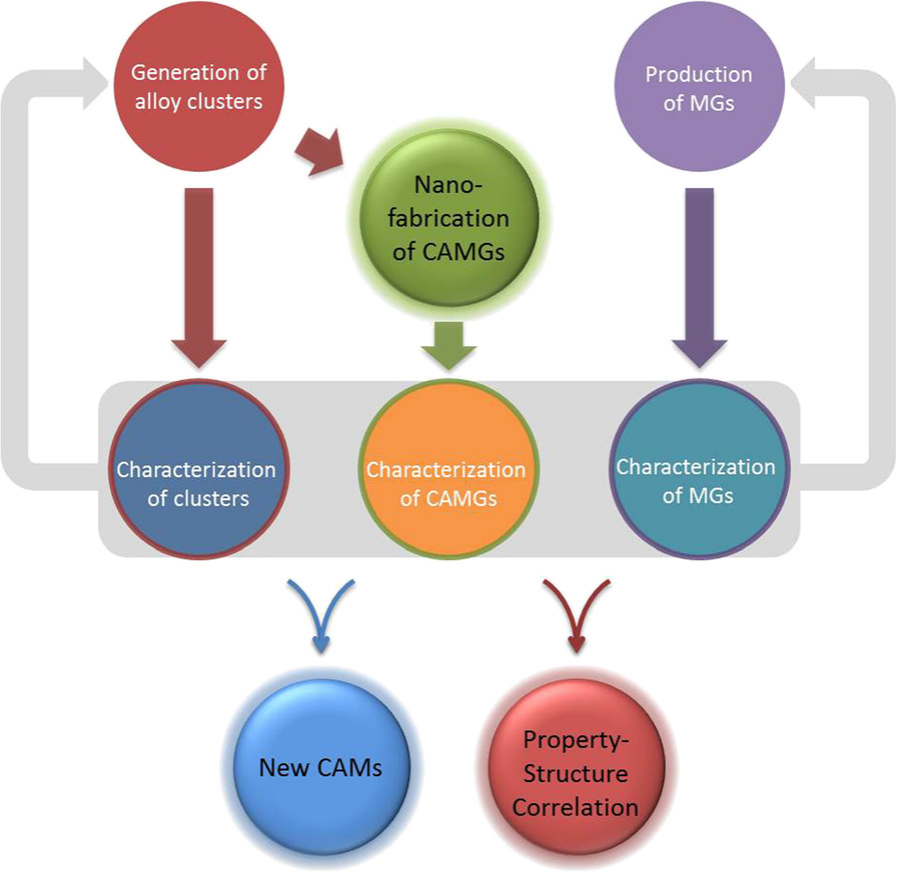
**The proposed hypothesis and its implications are summarized.** Nanofabrication of cluster-assembled metallic glasses followed by comparisons among properties of alloy clusters, CAMGs, and conventional metallic glasses can lead to understanding of the structure–property relation in amorphous materials and pave the way to the production of other cluster-assembled materials.

Figure 2 presents a graphical summary of the proposed idea and its implications. Performing such a delicate experiment, i.e., nanofabricating well-defined metallic glasses comprising size-selected metal clusters as building blocks, would shed new light on the atomic structure of metallic glasses. By combining the information achieved from the experiments proposed above, it would be possible to make a link between the structure of the cluster-assembled metallic glass (CAMG) and its properties. This has been a long-standing challenge to material scientists. Lack of this knowledge has restricted the design of new metallic glasses with specific properties to the costly and inefficient method of trial and error. The properties of the MG can also be related to those of the building blocks (metal clusters). The latter contains valuable information on CAMs, including but not limited to the stability of single clusters once in contact with other clusters and the interaction among clusters. This knowledge, on the other hand, can be very useful in designing new cluster-assembled materials.

## Abbreviations

CAM: Cluster-assembled materials; CAMG: Cluster-assembled metallic glass; MG: Metallic glasses.

## Competing interests

The author declares that he has no competing interests.
